# Low CXCL13 Expression, Splenic Lymphoid Tissue Atrophy and Germinal Center Disruption in Severe Canine Visceral Leishmaniasis

**DOI:** 10.1371/journal.pone.0029103

**Published:** 2012-01-05

**Authors:** Joselli S. Silva, Alan C. Andrade, Claudia C. Santana, Leina Q. Santos, Camila I. de Oliveira, Patrícia S. T. Veras, José Vassallo, Washington L. C. dos-Santos

**Affiliations:** 1 Fundação Oswaldo Cruz - Centro de Pesquisas Gonçalo Moniz, Salvador, Bahia, Brazil; 2 Escola Bahiana de Medicina e Saúde Pública - Biomedicina, Salvador, Bahia, Brazil; 3 Faculdade de Ciências Médicas – UNICAMP, Campinas, São Paulo, Brazil; University of Lausanne, Switzerland

## Abstract

Visceral leishmaniasis is associated with atrophy and histological disorganization of splenic compartments. In this paper, we compared organized and disorganized splenic lymphoid tissue from dogs naturally infected with *Leishmania infantum* assessing the size of the white pulp compartments, the distribution of T, B and S100^+^ dendritic cells, using immunohistochemistry and morphometry and the expression of CCR7 and the cytokines, CXCL13, lymphotoxin (LT)-α, LT-β, CCL19, CCL21, TNF-α, IL-10, IFN-γ and TGF-β, using by real time RT-PCR. The lymphoid follicles and marginal zones were smaller (3.2 and 1.9 times, respectively; Mann-Whitney, P<0.02) in animals with disorganized splenic tissue in comparison to those with organized splenic lymphoid tissue. In spleens with disorganized lymphoid tissue, the numbers of T cells and S100^+^ dendritic cells were decreased in the follicles, and the numbers of B cells were reduced in both the follicles and marginal zones. CXCL13 mRNA expression was lower in animals with disorganized lymphoid tissue (0.5±0.4) compared to those with organized lymphoid tissue (2.7±2.9, both relative to 18S expression, P = 0.01). These changes in the spleen were associated with higher frequency of severe disease (7/12) in the animals with disorganized than in animals with organized (2/13, Chi-square, P = 0.01) splenic lymphoid tissue. The data presented herein suggest that natural infection with *Leishmania infantum* is associated with the impairment of follicular dendritic cells, CXCL13 expression, B cell migration and germinal center formation and associates these changes with severe clinical forms of visceral leishmaniasis. Furthermore the fact that this work uses dogs naturally infected with *Leishmania infantum* emphasizes the relevance of the data presented herein for the knowledge on the canine and human visceral leishmaniasis.

## Introduction

Visceral leishmaniasis is endemic in many parts of the world and is a significant health problem in Asia, Africa, Europe and the Americas. Visceral leishmaniasis exists in both anthroponotic and zoonotic forms that are caused by *L. donovani* and *L. infantum*, respectively [1]. The main clinical signs of visceral leishmaniasis include fever, hepatomegaly, splenomegaly, anemia, leukopenia, hypergamaglobulinemia and emaciation [2], [3]. Death often results from cases of bleeding and from an increased susceptibility to secondary bacterial infection [3], [4]. Visceral leishmaniasis-associated lethality ranges from 6 to 12% [5]. Dogs, which are one of the most common companion animals, are susceptible to visceral leishmaniasis, and canine visceral leishmaniasis shares many of the above-mentioned aspects with the human disease. However, dogs present with a more frequent and severe skin parasitism [6]. Evidence suggests that dogs are the main urban reservoir for *L. infantum* [7], [8].

In addition to splenomegaly, visceral leishmaniasis consists of an initial stage of hyperplasia and subsequent atrophy of the splenic lymphoid tissue [9]. Recently, our group observed that dogs exhibiting markers of susceptibility to visceral leishmaniasis, including clinical signs of disease and active parasitism of the spleen, had higher frequencies of generalized white pulp atrophy, an absence of germinal centers and a lack of definition of white pulp compartments [10].

The spleen is a secondary lymphoid organ that is responsible for surveillance of the blood to protect against systemic infection [11]. Hyposplenic or asplenic patients have at least a 10 times higher risk of developing an overwhelming bacterial infection compared with the general population and an increased in susceptibility to infection by a variety of pathogens has been described in splenectomized dogs and human beings [12], [13], [14]. The protection provided by the spleen against infections results from innate mechanisms, such as the control of iron metabolism by the NRAMP protein family, and from the generation of an adaptive immune response against pathogens. The mechanisms responsible for splenic protection are based on the clearly defined structural organization of the spleen into compartments [11], [12], [15]. Autopsies of patients that have died from severe visceral leishmaniasis frequently show atrophy and a loss of splenic tissue structure [16] as well as bacterial infections, mainly of the skin, respiratory tract and middle ear [4]. Studies using a murine model of visceral leishmaniasis have shown that *Leishmania* infection causes the loss of cell populations in the spleen and impairs cell migration to the marginal zone and lymphoid follicles of the spleen, which together disrupt the structure of the spleen [17], [18], [19]. The loss of follicular dendritic cells and the actions of cytokines, such as TNF and IL-10, appear to be the main factors involved in this process [18], [20], [21], [22]. Other molecules, such as chemokines and cytokines from the lymphotoxin family, are also involved in the organization and maintenance of splenic structure [23], [24], [25].

Studies using a murine model of experimental infection have provided much data on the pathways involved in the disruption of the splenic microenvironments during visceral leishmaniasis. Although these studies have defined pathogenic mechanisms of leishmaniasis infection, the experimental models use artificial conditions, such as the size of the inoculum, the origin and differentiation stage of the parasite and possibly the number of inocula. Hence, some of the conclusions that have emerged from the study of experimental infections still require confirmation during natural infection. In this work, we investigated the distribution of the main cell populations and cytokines potentially responsible for the organization of the splenic white pulp in dogs naturally infected with *L. infantum*. Furthermore, we compared the organized and disorganized spleen histologies of these animals. Canine visceral leishmaniasis is a disease worthy of study for veterinary and public health reasons [26]. Naturally acquired canine visceral leishmaniasis may also represent a model of human disease, as both dogs and humans are subject to similar conditions for infectious transmission in endemic areas and develop diseases with many identical signs (reviewed above). Hence, this study provides a unique contribution in terms of the confirmation and refinement of the conclusions obtained from the studies of experimental models of visceral leishmaniasis.

Our aim for this study was to test the hypotheses raised by studies of murine visceral leishmaniasis in a naturally infected animal that is involved in the transmission of the disease in endemic areas. An understanding of the pathways involved in the structural disruption of splenic compartments in dogs naturally infected with *L. infantum* may contribute to the development of therapeutic strategies for the treatment of severe forms of visceral leishmaniasis in dogs and humans.

## Materials and Methods

### Ethics statement

All procedures involving animals were conducted in accordance with Brazilian Federal Law on Animal Experimentation (Law 11794) [27] (http://www.planalto.gov.br/ccivil_03/_ato20072010/2008/lei/l11794.htm), with the Oswaldo Cruz Foundation guidelines for research with animals (http://sistemas.cpqam.fiocruz.br/ceua/hiceuaw000.aspx) and with the manual for the surveillance and control of visceral leishmaniasis [28]. This study was approved by the ethics committee for the use of animals in research (CPqGM-FIOCRUZ, Ceua, license N.040/2005).

### Animals

Splenic samples from 25 dogs were used for this study. The specimens were obtained from the canine leishmaniasis tissue sample archive of the Pathology and Bio-Intervention Laboratory at the Gonçalo Moniz Research Center of FIOCRUZ in Salvador, BA, Brazil. The samples were from stray dogs of different breeds and estimated ages that were collected from the streets of Jequié (Bahia State, Brazil, an endemic area for visceral leishmaniasis) between 2004 and 2008. This was done in collaboration with the Endemic Diseases Surveillance Program of the State Health Service as part of a program for the surveillance and control of visceral leishmaniasis. All the animals were clinically examined by at least two Veterinarians using a clinical chart containing the following parameters: gender, emaciation, anemia, dermatitis, alopecia, conjunctivitis, onychogryphosis, increased lymph node size, splenomegaly and apathy. The animals were grouped into the following categories, according the reported clinical signs suggestive of visceral leishmaniasis: asymptomatic (with none clinical signs), oligosymptomatic (with no more than three clinical) and polysymptomatic (with more than three clinical signs). The presence of anti-*Leishmania* antibodies in the serum was determined by ELISA, and the cellular immune response against *L. infantum* antigens was detected by the leishmanin skin test (LST). Immediately following euthanasia, spleen aspirates were collected for culture, and spleen fragments were collected and frozen in liquid nitrogen for molecular biology studies or were fixed in formalin and embedded in paraffin for morphological studies. For determination of cytokine expression in the spleens of uninfected animals (negative ELISA, LST and PCR for *Leishmania* infection) with organized spleen structure, six animals collected from the streets of Jequié in the same period and conditions described above were used. The technical details of the anti-*Leishmania* ELISA, the LST and the splenic culture for *Leishmania* isolation have been reported elsewhere [29]. As tissue specimens were not always available from each animal for each of the studies, not all animals were subjected to each test described below.

### Spleen samples and histological analysis

Three to four mm thick slices of splenic tissue were collected by perpendicular section to the surface of the spleen in the middle part of the organ. The tissue slices were fixed in formalin and embedded in paraffin. Hematoxylin- and eosin-stained splenic tissue sections 4.0 to 5.0 µm thick were examined by optical microscopy. The samples were divided into two groups according to the degree of structural organization of the splenic white pulp using criteria previously described by Santana and colleagues (2008) [10]. The two groups were as follows: (a) animals with well-organized splenic lymphoid tissue and (b) animals with moderately or extensively disorganized splenic lymphoid tissue.

### Immunophenotyping of spleen cells

The immunophenotyping of spleen cells was done using immunohistochemistry as described by Ruiz and collaborators [30] with some modifications. Briefly, serial spleen sections were placed on poly-L-lysine (Sigma-Aldrich, United States)-coated slides and dewaxed. Antigen retrieval was performed with citrate buffer at pH 6.0 (for the anti-S100 and anti-CD3 antibodies) or tris-EDTA at pH 8.9 (for the anti-CD79a and anti-Ki67 antibodies). For the remaining steps, the Novolink Max Polymer (Novocastra, United Kingdom) system was used, and the protocol provided by the manufacturer was followed. The slides were incubated for 1 hour at 37°C with primary anti-CD3 (clone F7.2.38), anti-Ki67 (clone MIB-1) or anti-CD79α (clone HM57) antibodies and a polyclonal anti-S100 antibody from Dako (Carpinteria, CA). In some experiments, the positive controls consisted of human tissues known to be reactive with each marker. As a negative control, sections were incubated with buffer only or with an immunoglobulin of the same isotype but different specificity as the test antibody. After staining, the sections were counterstained with hematoxylin, and the immunolabeling was evaluated microscopically.

### Morphometry

**Figure 1:**
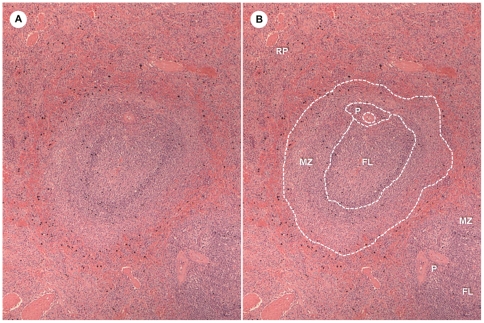
Splenic white pulp compartments defined for morphometric estimative. **A** – Dog spleen section showing the red pulp and the white pulp compartments; **B** – white pulp compartments estimated by morphometry. RP = red pulp, MZ = marginal zone, FL = follicle, P = PALS.

Images of spleen sections stained with different antibodies were captured at a resolution of 1,280×1,024 pixels using an Evolution LC digital video camera system (Media Cybernetics, United States) coupled to an optical microscope (CX41, Olympus, Japan). The red pulp, white pulp and various white pulp compartments, including the periarteriolar lymphocyte sheath (PALS), lymphoid follicles and marginal zone (MZ), were morphometrically estimated using Image-Pro Plus version 6.0 software (Media Cybernetics, United States). The boundaries of each compartment were defined using hematoxylin and eosin-stained sections and confirmed using S100 protein-stained spleen sections. The different compartments of the white pulp of the spleen were identified according histological descriptions of splenic structure [15], [31] (see Fig. 1). For the cell counts, random and non-overlapping areas in the red pulp and white pulp compartments were selected. To minimize error for the measurements of the sizes of the white pulp compartments, the following criteria were used: (1) the regions associated with the five largest follicles in each section were selected for analysis of lymphoid follicles and marginal zone and (2) only arterioles represented in transversal section were used for analysis of the PALS. Serial sections of at least one representative slice of the spleen of each dog were used in the study. The mean measurement obtained from each compartment of the white pulp was used for further estimates of the cell population density (cell population density = number of cells/defined area) in each compartment and the total number of cells in a cross section of the compartment (average number of cell per compartment area = cell population density in the compartment×the compartment area). The measure of the cell density represents how closely packed the cell population is within the compartment, and the total number of cells per cross section of the compartment is influenced by the size of the compartment and represents the size of the cell population within a particular compartment of the spleen.

### Cytokine expression analysis by real-time RT-PCR

Splenic expression of CCR7 and the cytokines, CCL19, CCL21, CXCL13, LTα, LTβ, IL10, IFNγ, TGFβ and TNF, from 22 *Leishmania*-infected dogs was determined using RT-PCR. Of the 22 dogs, 12 had organized splenic lymphoid tissue and 10 had disorganized lymphoid tissue. Six non-infected animals with organized splenic structure were used as a control for cytokine expression in the spleen of non-*Leishmania*-infected animals. Frozen spleen fragments were thawed and macerated in Trizol (Invitrogen Life Technologies, United States) for total RNA extraction according to the manufacturer's recommendations. Contaminating DNA present in the RNA extract was removed by DNase digestion using the RNeasy kit (Qiagen, Germany). After the synthesis of the complementary DNA (cDNA) using Superscript II reverse transcriptase (Invitrogen Life Technologies, United States), real-time PCR was performed in a 20 µl reaction volume that included 10 µl of SYBR1 Green PCR Master Mix (Applied Biosystems, United States), 200 nM of each primer, 5 µl of cDNA and nuclease free water for the remaining volume. For each gene of interest, the reaction was performed in duplicate with triplicate negative controls on each plate. The primer pair sequences and the thermal cycle conditions for each gene amplification are shown in Table 1. A cycle threshold value was calculated for each sample. Triplicate standard curves were constructed by serial dilution (1∶5) from a concentrated pool of cDNA samples as previously described [32]. All reactions were performed in optical 96-well reaction plates using the ABI Prism 7500 system (Applied Biosystems, United States). Cytokine mRNA concentrations from each sample were calculated based on the standard curve and were normalized to the concentration of the housekeeping gene, 18S rRNA. In addition to DNase digestion of potentially contaminating DNA present in the RNA extract the usual technical caries were taken in order to avoid native DNA amplification [33]. Furthermore, the specificity of the amplification of genes of interest was confirmed by genomic sequencing of the PCR products. The sequencing was performed using the DNA sequencing services at CDTS-FIOCRUZ (http://plataformas.cdts.fiocruz.br/site/home.aspx).

**Table 1:** Primer sequences and qRT-PCR conditions for determination of cytokine expression in the spleens of dogs with visceral leishmaniasis.

Primer	Forward primer (5′ – 3′)	Reverse primer (5′ – 3′)	Genbank reference sequence	Annealing temperature
18S	CACGGCCGGTACAGTGAAAC	CCCGTCGGCATGTATTAGCT	M27358	60°
CCL19	GTAGACTGCCTGCCGTTGTGTTCA	ACTGCTGTGGCCCTTGTTCTTTG	NM_001005256.1	60°
CCL21	GCACAGGACTGTTGCCTCAA	CGGTAGCTGCGGACAACCT	NM_001005258	55°
CCR7	GGTGGTGGCTCTCCTTGTCATTTT	AGTTCCGCACGTCTTTCTTGAAGC	XM_548131.2	55°
CXCL13	GGGTGCCCAAAAAGAGAAATC	GATGGGAGGGTTCAAGCATACA	XM_845089.1	55°
LT-α	GAGACCCCAGCATCCAGAAC	AAGGCACGATCCGTGTTTG	XM_843793.1	60°
LT-β	CGGCTGGGAGGCGAAGAAAG	GGTAGGCGACGTGACAGTAGAGGT	NM_001033510.1	60°
IL10	CCACGACCCAGACATCAAGAA	ACAGGGAAGAAATCGGTGACA	U33843	60°
TGF-β	CCACTGTTCCTGTGACAGCAA	GTCGGTTCATGCCATGAATG	L34956	60°
TNF-α	AGCAAACCCCGAAGCTGAG	CGGCACTATCAGCTGGTTGTC	S74068	60°
IFN-γ	AAGGAAGACATGCTTGGCAAG	CCTGCAGATCGTTCACAGGAA	NM_001003174	60°

### Real-time PCR for the detection of *Leishmania* DNA

Results of conventional qualitative PCR for *Leishmania* DNA detection was available for the animals collected in the year of 2004 (five with organized and three with disorganized splenic lymphoid tissue). To detect parasite DNA in frozen spleen samples of the animals collected in the years 2006 and 2008 (seven with organized and eight with disorganized splenic lymphoid tissue) a quantitative real time PCR technique was used. Both techniques are briefly described below.

#### DNA extraction

Frozen spleen fragments were macerated in 400 µl of lysis buffer (50 mM Tris-HCl, EDTA, pH 8.5), centrifuged for 5 min at 12,000 *g*. The pellet was resuspended in 40 µl of SDS (10%) and 4 µl of proteinase K (20 mg/ml), homogenized and incubated for 2 hours at 42°C. Extraction with phenol-chloroform and isoamyl alcohol (Invitrogen Life Technologies, United States) was performed and followed by precipitation with 800 µl of ethanol 100% and 40 µl of sodium acetate (3 M). The mixture was incubated overnight at −20°C then centrifuged at 12,000×g for 5 min. The pellet was washed with 1 ml of ethanol 70% and centrifuged at 12,000×g for 5 min, dried for several minutes, rehydrated in water (100 µl) and stored at −20°C.

#### Conventional qualitative PCR for *Leishmania* DNA detection

PCR was performed with primers 5′-GGG(G/T)AGGGGCGTTCT(G/C)CGAA-3′ and 5′-(G/C)(G/C)(G/C)(A/T)CTAT(A/T)TTACACCAACCCC-3′, which target the amplification of the 120-bp conserved region of the *Leishmania* kDNA minicircle of all *Leishmania* species. A reaction mixture was prepared containing 50 mmol/l KCl, 10 mmol Tris–HCl (pH 8.0), 0.2 mmol/l each deoxyribonucleotide (Invitrogen), 1 µmol/l each primer, 1.25 units of Taq polymerase (Invitrogen) and 2.5 µl of splenic DNA sample in a final volume of 25 µl. The PCR conditions were as follows: denaturation at 94°C for 3 min, followed by 30 cycles of 94°C for 30 s, 55°C for 30 s and 94°C for 45 s with a final extension of 72°C for 10 min. The amplification reactions were analyzed by agarose gel electrophoresis, followed by ethidium bromide staining and visualization under UV light. DNA from the reference *L. braziliensis* strain MHOM/BR/75/2904 was used as a positive control.

#### Real time PCR for *Leishmania* DNA detection

This assay was performed using a previously described technique [34] targeting a SSU rRNA gene sequence. The amplifications were performed in a final volume of 25 µl that consisted of 5 µl of splenic DNA diluted in deionized water to 150 ng/µl and 20 µl of the PCR mixture, which included 12.5 µl of the Universal Mastermix (Perkin-Elmer Applied Biosystems), a forward primer (5′-AAGGTCAAAGAACAAGGCCAAG-3′) at a final concentration of 900 nM, a reverse primer (5′-GCATCGGAGTCGG-3′) at 300 nM and a fluorogenic probe (5′-AGGAGCGTGTCCCCGTGGAGG-3′) containing a FAM reporter molecule at the 5′ end and a TAMRA quencher at the 3′ end (LEIP-probe) (Perkin-Elmer Applied Biosystems) at a final concentration of 200 nM. A standard curve was generated using serial dilutions of *L. infantum* DNA from 10^6^ to 10^−1^ parasites/mL, and each dilution was performed in triplicate. The amplifications were performed in triplicate for each sample and for the negative control using an ABI Prism 5900 sequence detection system (Perkin-Elmer Applied Biosystems). The standard curve was generated by plotting the Ct values against the standardized parasite concentrations.

#### Expression and analysis of the results

Numerical data are shown as tables and graphs and represent the absolute values, means, medians or proportions as stated. The statistical significance of the differences between groups was tested using the Mann-Whitney test. For comparisons involving more than two groups Kruskal-Wallis test was used. For comparisons involving proportions, the Chi-square test with the Yates' correction or Fisher's exact probability test were used as recommended [35]. The level of significance was established at P<0.05.

## Results

The main characteristics of the animal groups used in this study are summarized in Table 2. Clinical signs of visceral leishmaniasis were more frequent in the animals with disorganized splenic lymphoid tissue (7/12 animals were polysymptomatic) than in animals with organized tissue (only 2/13 animals were polysymptomatic; Chi-square test, P = 0.01). There were no significant differences in the gender, in the distribution of positive tests for *Leishmania* infection or parasite burden in the spleen, between the groups. However, 50% of the animals with disorganized splenic lymphoid tissue had *Leishmania* burdens above 35,294 parasites per 100 mg of spleen, while most of the animals with organized splenic lymphoid tissue had *Leishmania* burden of 3,514 or less parasites per 100 mg of spleen (table 2).

**Table 2:** General characteristics of the animals used in the study.

	Histological structure of spleen		
Positive parameters	Organized(n = 13)	Disorganized(n = 12)	Significance
Gender	F = 7/M = 6	F = 5/M = 7	P = 0.70^a^
Clinical category^b^			
Oligosymptomatic	11	5	
Polysymptomatic	2	7	P = 0.01^a^
Positive test for *Leishmania* infection:			
Spleen culture	6/13 (46%)	6/12 (50%)	P = 1.00^a^
Serology	8/13 (62%)	8/12 (67%)	P = 1.00^a^
LST	2/13 (15%)	1/12 (8%)	P = 1.00^a^
PCR	12/12 (100%)	11/11 (100%)	P = 1.00^a^
Parasite/100 µg of spleen (quantitative real time PCR)^c^			
25% percentile	1.8×10^3^	6.2×10^2^	
Median	3.5×10^3^	3.5×10^4^	P = 0.96^d^
75% percentile	1.9×10^5^	3.4×10^6^	
At least one test positive	13/13 (100%)	12/12 (100%)	NT^e^

a Fisher's exact probability test;

b With 1–3 (oligosymptomatic) or more than 3 (polysymptomatic) of the following clinical signs: emaciation, anemia, dermatitis, alopecia, conjunctivitis, onychogriphosis, increased lymph node size, splenomegaly and apathy. Detail are given in the text;

c Quantitative PCR estimative only available for eight more recently collected animals of each group; Quantitative PCR was not available for 5 animals with organized and for 3 animals with disorganized splenic lymphoid tissue;

d Mann-Whitney test.

e Not tested.

### Distribution of red pulp and white pulp compartments in disorganized spleens

In animals with organized splenic lymphoid tissue, the proportion of white pulp to the total area of the spleen section was twice as large (11.5±3.7%) as that observed in animals with disorganized splenic lymphoid tissue (5.7±4.3%; Mann-Whitney; P<0.03) (Table 3). However, the differences in the size of the white pulp compartments between the two animal groups were not uniform; the lymphoid follicles and the marginal zone were smaller in the animals with disorganized lymphoid tissue than in animals with organized tissue, but the size of the PALS was not significantly different between the groups (Table 3).

**Table 3:** Estimation of the areas represented by the different compartments of the spleen in animals with organized or disorganized splenic histological architecture.

Spleen	Histological structure of spleen			
compartments	Organized	Disorganized	Ratio^a^	Significance^b^
Red pulp^c^	88.5±3.7	94.3±4.3	0.9	P<0.03
White pulp^c^	11.5±3.7	5.7±4.3	2.0	P<0.03
PALS^d^	6374±2814	6836±2602	0.9	P = 0.67
Follicle^d^	133870±53950	38921±16823	3.4	P<0.01
Marginal zone^d^	283924±169036	151241±83286	1.9	P<0.02

a Organized/disorganized ratios;

b Unpaired t test or Mann-Whitney test when recommended.

c Proportion (%) of the splenic tissue represented by the specific compartment either red or white pulp in the histological sections;

d Mean and sd of the compartment (PALS, follicle or marginal zone) area expressed in mµ^2^.

### Distribution of leukocyte populations in organized and disorganized spleens

**Figure 2:**
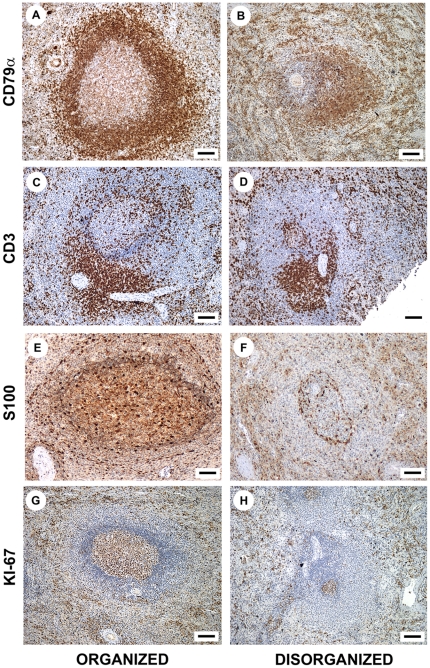
Leukocyte populations in organized and disorganized spleens. Distribution of CD79α^+^ B and CD3^+^ T lymphocytes, S100^+^ dendritic cells and Ki-67^+^ proliferating cells in the spleens of dogs infected with ***L. infantum*** with and without disruption of splenic lymphoid tissue structure (figures **A**, **B**, **C**, **D**, **G** and **H**, bar = 70 µm; figures **E** and **F**, bar = 50 µm).

The distribution of leukocyte populations was similar between the spleens of dogs with organized and disorganized splenic lymphoid tissue. CD3^+^ cells (T lymphocytes) were preferentially found in the PALS and were more diffusely spread within the lymphoid follicles, marginal zone and red pulp. In the red pulp, T lymphocytes were distributed in small clusters (Fig. 2 C, D). CD79α^+^ cells (B lymphocytes) were predominantly observed in the lymphoid follicles of both animal groups, although the germinal centers and the mantle regions were not always evident in animals with disorganized splenic lymphoid tissue. In the red pulp, B lymphocytes were preferentially distributed in small clusters (Fig. 2 A, B). S100^+^ dendritic cells [36], [37] were predominantly found to line the edges of the white pulp compartments but were also found in the interior of the compartments (Fig. 2 E, F). Some of these cells were also dispersed in the red pulp. Ki-67^+^ (proliferating) cells were present predominantly in the germinal center of the lymphoid follicle and distributed in aggregates throughout the mantle and marginal zone. Proliferating cells were more sparsely distributed in the red pulp of both groups (Fig. 2 G, H).

Although we did not observe substantial qualitative changes in the distribution patterns of leukocyte subsets in the compartments of the disorganized spleens, there was a decrease in the number of cells in some compartments. Hence, we used morphometry to compare the density and the number of cells in the different spleen compartments between the animal groups.

### Density of different leukocyte populations in disorganized spleens

The densities of CD79α^+^ B lymphocytes (1.4±0.8×10^4^ cells/mm^2^) and proliferating (Ki-67^+^) cells (3.0±2.2×10^3^ cells/mm^2^) were lower in the lymphoid follicles of dogs with disorganized splenic lymphoid tissue than in those of dogs with organized splenic tissue (3.7±0.9×10^4^ B lymphocytes/mm^2^ and 7.6±1.7×10^3^ Ki-67^+^ cells/mm^2^; Mann-Whitney test; P<0.01). There were no other differences observed between the animal groups in the densities of leukocyte subsets in the splenic compartments.

### Estimation of the number of leukocytes per cross sectional area of splenic compartments

**Figure 3:**
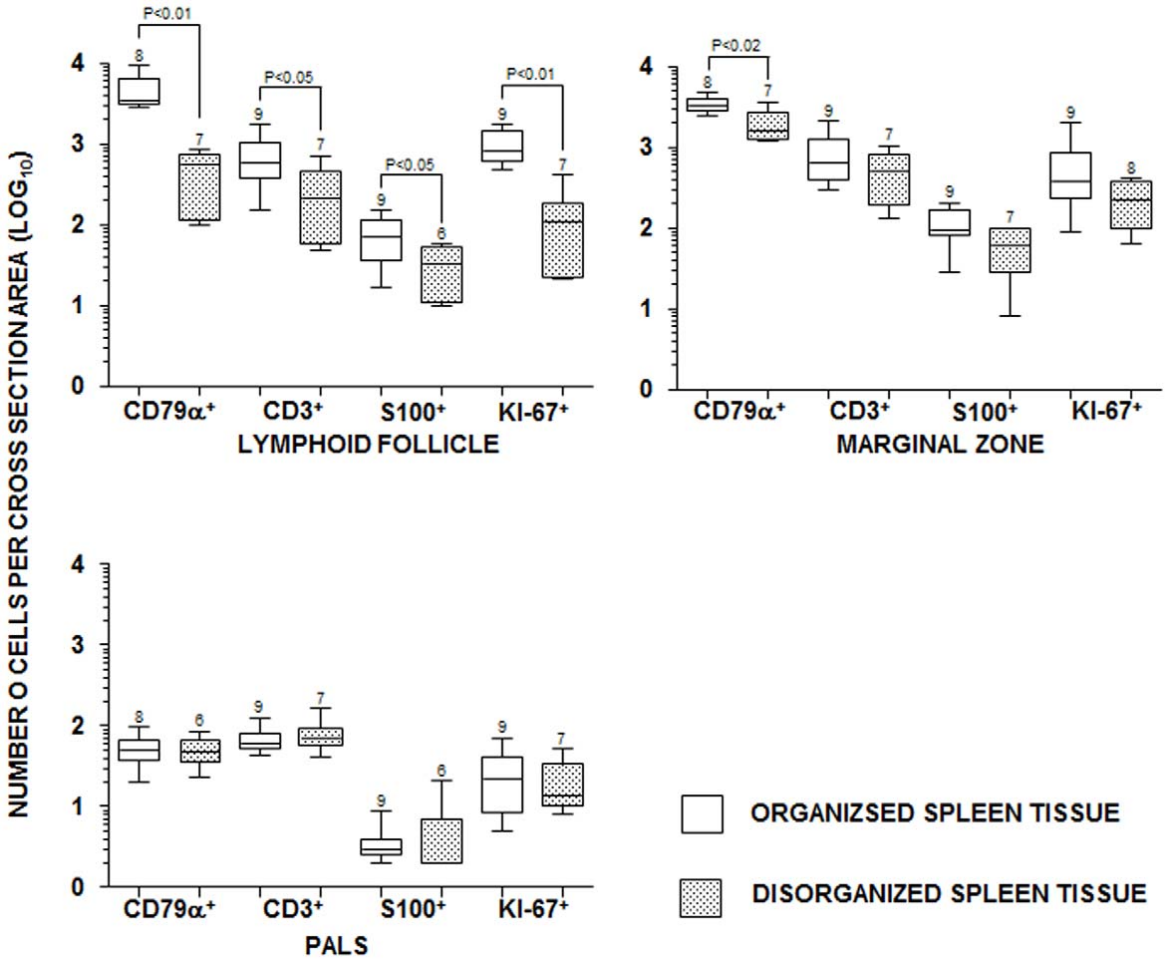
B cell, T cell, dendritic and proliferating cells number in organized and disorganized spleens. Estimation of the average number of CD79α^+^ B and CD3^+^ T lymphocytes, S100^+^ dendritic cells and Ki-67^+^ proliferating cells in cross sections of the splenic compartments of dogs infected with *L. infantum* with and without disruption of splenic lymphoid tissue structure. Box and whisker plots represent median, 25% and 75% percentiles and ranges. Y-axis scale is in Log_10_. The number of animals used in each estimative is shown on the top of the up range bar. P values (Mann-Whitney test) only shown for statistically significant differences.

The average numbers of CD79α^+^ B cells (477±288), T cells (289±243), S100^+^ DCs (33±20) and proliferating cells (130±137) were lower in the splenic lymphoid follicles of animals with disorganized lymphoid tissue than in the splenic lymphoid follicles of animals with organized tissue (B cells = 4,724±2,362, P<0.01; T cells = 725±499, P = 0.04; S100^+^ DCs = 77±44, P = 0.04; proliferating cells = 1,008±462, P<0.01; Mann-Whitney test; Fig. 3). The average number of CD79^+^ B lymphocytes (2.0±0.9×10^3^) was also lower in the marginal zones of animals with disorganized tissue than in those of animals with organized tissue (3.4±0.7×10^3^; Mann-Whitney test; P = 0.01) (Fig. 3). There were no additional differences between groups in the number of cells in other splenic compartments.

### Cytokine and CCR7 expression in disorganized splenic tissue

**Figure 4:**
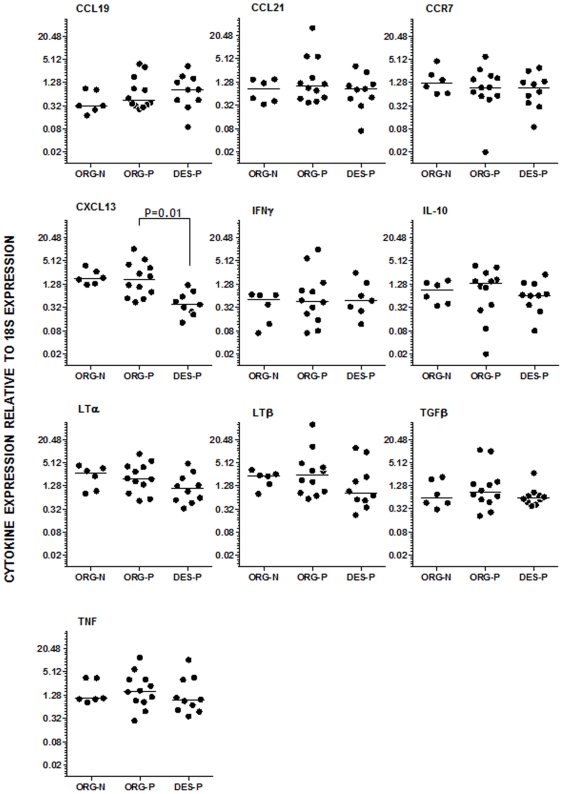
Cytokine expression in organized and disorganized spleens. The gene expression of cytokines and CCR7 in the splenic compartments of stray dogs uninfected with organized splenic white pulp (ORG-N), infected with *L. infantum* with (DES-P) and without (ORG-P) disruption of splenic lymphoid tissue structure. Horizontal lines represent the median of the estimates from RT-PCR, and the values are relative to 18S gene expression. Each dot corresponds to a different animal with 12 organized (12) or disorganized (9) splenic lymphoid tissue. P values (Kruskal-Wallis test) only shown for statistically significant differences.

As there is very limited information on the cytokine expression pattern in the canine spleen, we used a group of animals from the same endemic area that were negative for *Leishmania* infection for serology, culture and PCR of spleen aspirates as a control for the levels of cytokine expression. The gene expression of each of the tested cytokines and CCR7 relative to 18S expression is shown in Figure 4. The gene expression of CXCL13 was lower in the *Leishmania*-infected animals with disorganized splenic lymphoid tissue (0.5±0.4) than in the *Leishmania*-infected animals with organized splenic lymphoid tissue (2.7±2.9) or the control animals (2.1±1.0). There were no statistically significant differences in the gene expression of the other cytokines examined (CCL19, CCL21, IFN-γ, IL10, LTα, LTβ, TGF-β and TNF) or CCR7 between the *Leishmania*-infected groups. Furthermore, no differences were observed between infected animals with organized splenic tissue and control animals (Fig. 4).

## Discussion

The atrophy of lymphoid tissue and the disorganization of splenic microenvironments have been observed during infection with *Leishmania* as well as with other pathogens [9], [18], [38]. In a previous study, we found that these pathological changes in the spleen were more frequent in dogs with clinical and laboratory patterns of susceptibility to visceral leishmaniasis than in those without evidence of susceptibility to the disease [10]. In this work, we studied two groups of naturally infected dogs with distinct splenic histological organization. Dogs with a structural disorganization of the spleen had more severe visceral leishmaniasis than did animals without splenic structural disorganization. This observation on the relationship between clinical disease and disruption of splenic structure confirms data from studies on human leishmaniasis [16] and concur with our previous observations associating disorganization of the splenic lymphoid tissue in dogs and susceptibility to the disease [10].

Changes to the spleen that vary from hyperplasia to atrophy have been described for humans and dogs with visceral leishmaniasis [9], [16], [39], [40], [41]. The sequential occurrence of hyperplasia followed by atrophy was demonstrated by Veress and collaborators [9] using an experimental model of visceral leishmaniasis in hamsters. Such atrophy and disappearance of splenic B cell areas coincided with the later and more severe stages of the disease. In our previous work, we found that animals with active infection and cellular responses to *Leishmania* antigens, represented by positive leishmanin skin tests, predominantly displayed hyperplasia of the splenic white pulp, whereas atrophy and the disruption of splenic lymphoid architecture were more frequently seen in animals with active infection but negative leishmanin skin tests [10]. In this work, we showed that although the entire white pulp was reduced in size, the follicles and the marginal zone were the compartments of the white pulp most severely affected. These changes in the white pulp were associated with decreased numbers of T and dendritic cells in the follicles and B cells in the follicles and marginal zones. This pattern of cell depletion indicates a disruption to the follicles, with the most striking change observed in the B cell population and impairment of germinal center formation. B cells are the main constituents of lymphoid follicles, and various factors contribute to their entry, maintenance and proliferation in the splenic lymphoid compartments. The organization of the lymphoid follicles requires CXCL13-mediated B cell migration into the follicle and the arrest of these cells in proximity to follicular dendritic cells [42]. CXCL13 also induces the membrane expression of LTα1β2 by B cells, which in turn promotes follicular dendritic cell development and CXCL13 expression to create a positive feedback network [11]. In this work, we found that CXCL13 expression was decreased in animals displaying disruption and atrophy of lymphoid follicles and reduction or absence of follicular germinal centers. It has been shown that disrupted expression of the gene for CXCL13 or its B cell surface receptor, CXCR5, results in impaired B cell migration into the follicle and germinal center formation following antigen exposure [43], [44]. Furthermore, the slight and non-significant decrease in the expression of LTα and LTβ in animals with disorganized splenic histology suggests that CXCL13 may be the main cytokine involved in this process. In this study, we also demonstrated decreased numbers of follicular dendritic cells in the follicles of animals with disorganized white pulp structure. The combination of the decreased follicular dendritic cell number and the reduced CXCL13 expression may suggest a causal association between each phenomenon; follicular cell damage may result in lower CXCL13 production, which then may lead to deficient B cell migration and follicular atrophy.

Certain pathological processes may be responsible for the disruption of this CXCL13-dependent cross-stimulation loop between B cells and follicular dendritic cells during visceral leishmaniasis. Smelt and collaborators [17] have proposed that factors released by amastigote-infected macrophages infiltrating the lymphoid follicles are responsible for follicular dendritic cell destruction. In our study, although most of the animals presented evidence of spleen parasitism by PCR or spleen culture, amastigote-containing macrophages were less frequently observed by optical microscopy than in the study by Smelt and collaborators [17]. However, in the present study, naturally infected dogs with parasite burdens lower than experimental model hosts were studied. Therefore, the continuous but less severe destruction of follicular dendritic cells, which eventually leads to follicle disruption, may also be caused by infected macrophages. However, specific characterization of a putative follicular dendritic cell-damaging molecule has not been performed. Engwerda and collaborators (2002) also found a decrease in lymphocyte migration into the marginal zone that was associated with a reduction in the population of marginal zone macrophages in mice infected with *L. donovani*. Such impairment of lymphocyte migration may affect the structure of the marginal zone and the lymphoid follicle, and most of these changes were dependent upon TNF expression [18]. Although the animals with disrupted splenic lymphoid tissue structure in this study had smaller marginal zones and a lower number of B cells associated with this compartment, there were no differences in TNF expression between the animal groups. Increased levels of TNF have been observed in human and murine experimental models of visceral leishmaniasis [45], [46]. Melby and collaborators (2001) [46] found an initial increase and subsequent decrease in TNF production in the spleens of hamsters experimentally infected with *Leishmania donovani*. Hence, we cannot exclude the possibility that periods of high TNF production take place during the natural course of canine visceral leishmaniasis. Another potential cause of the lymphoid tissue disruption seen in some of the animals is the presence of co-infections with viruses or other pathogens that contribute to the disorganization of splenic structure. For example, a similar pattern of hyperplasia and atrophy followed by disorganization of lymphoid follicles has been described for viral infections in humans and experimental animals [38], [47]. A serological survey performed by our group of street dogs showed a high prevalence of infection with *Erlichia canis* and *Anaplasma* (unpublished data). Therefore, other infections are common in these animals, and we cannot exclude the possibility that some of these infections may contribute to the disruption of the splenic lymphoid tissue structure.

Although more extreme values of IFNγ, IL10 and TNF expression were observed in infected animals, we found no statistically significant differences in the expression of any other tested cytokines, apart from CXCL13 between the infected and uninfected group of animals. We cannot exclude the possibility that other systemic infections, also prevalent in stray dogs, as mentioned above, may be modulating cytokine expression in this non-*Leishmania*-infected group. Lage and colleagues [48] found higher levels of IFN-γ expression by splenocytes of *Leishmania*-infected than in non-infected dogs, using a mixed group of stray and domiciled dogs, previously vaccinated against parvovirosis, leptospirosis, distemper, parainfluenza and hepatitis and treated for intestinal helminthic infections. However, these authors did not find statistically significant differences in the frequency of IL10, IL12, IFNγ or TNF between the animals of the uninfected and *Leishmania*-infected groups.

Finally, it has been proposed that the disorganization of splenic architecture may be associated with an impaired retention of activated B cells in the follicles, with the release of these cells into the circulation and with greater plasma cell production [47]. In fact, plasma cells predominate in the inflammatory infiltrates observed in visceral leishmaniasis [16], [40], [41] and we have previously shown that the frequency of plasma cells is increased in the spleens of dogs naturally infected with *Leishmania* [10]. Nevertheless, the structural disorganization of the spleen secondary to leishmaniasis and possibly due to co-infection may affect the microenvironments of the spleen that are necessary for B cell activation, memory B cell homing and the generation of humoral immune responses to blood-borne antigens. In fact, splenic atrophy and disorganization as well as impaired host responses to bacterial infection have been found in patients during the late stages of severe visceral leishmaniasis [4], [16]. A study on the potential association between splenic disorganization and the emergence of bacterial infections in dogs with severe forms of visceral leishmaniasis is now underway in our laboratory.
